# Lipoprotein (a) predicts recurrent worse outcomes in type 2 diabetes mellitus patients with prior cardiovascular events: a prospective, observational cohort study

**DOI:** 10.1186/s12933-020-01083-8

**Published:** 2020-07-09

**Authors:** Yan Zhang, Jing-Lu Jin, Ye-Xuan Cao, Hui-Wen Zhang, Yuan-Lin Guo, Na-Qiong Wu, Cheng-Gang Zhu, Ying Gao, Qi Hua, Yan-Fang Li, Rui-Xia Xu, Jian-Jun Li

**Affiliations:** 1 State Key Laboratory of Cardiovascular Disease, FuWai Hospital, National Center for Cardiovascular Diseases, Chinese Academy of Medical Sciences, Peking Union Medical College, BeiLiShi Road 167, Beijing, 100037 China; 2 Department of Cardiology, Xuanwu Hospital, Capital Medical University, Beijing, China; 3 Department of Cardiology, Beijing Anzhen Hospital, Capital Medical University, Beijing, China

**Keywords:** CAD, HBA1c, Lp(a), Recurrent CVEs, T2DM

## Abstract

**Background:**

Merging studies have reported the association of lipoprotein(a) [Lp(a)] with poor outcomes of coronary artery disease (CAD) in patients with type 2 diabetes mellitus (T2DM). However, the prognostic importance of Lp(a) for recurrent cardiovascular events (CVEs) is currently undetermined in patients with T2DM and prior CVEs.

**Methods:**

From April 2011 to March 2017, we consecutively recruited 2284 T2DM patients with prior CVEs. Patients were categorized into low, medium, and high groups by Lp(a) levels and followed up for recurrent CVEs, including nonfatal acute myocardial infarction, stroke, and cardiovascular mortality. Kaplan–Meier, Cox regression and C-statistic analyses were performed.

**Results:**

During 7613 patient-years’ follow-up, 153 recurrent CVEs occurred. Lp(a) levels were significantly higher in patients with recurrent CVEs than counterparts (20.44 vs. 14.71 mg/dL, p = 0.002). Kaplan–Meier analysis revealed that the event-free survival rate was dramatically lower in high and medium Lp(a) groups than that in low group irrespective of HBA1c status (< 7.0%; ≥ 7.0%, both p < 0.05). Furthermore, multivariate Cox regression models indicated that Lp(a) was independently associated with high risk of recurrent CVEs [HR(95% CI): 2.049 (1.308–3.212)], such data remains in different HBA1c status (HR(95% CI): < 7.0%, 2.009 (1.051–3.840); ≥ 7.0%, 2.162 (1.148–4.073)). Moreover, the results of C-statistic were significantly improved by 0.029 when added Lp(a) to the Cox model.

**Conclusions:**

Our data, for the first time, confirmed that Lp(a) was an independent predictor for recurrent CVEs in T2DM patients with prior CVEs, suggesting that Lp(a) measurement may help to further risk stratification for T2DM patients after they suffered a first CVE.

## Background

It has been demonstrated that atherosclerotic cardiovascular disease (ASCVD) is the leading causes of morbidity and mortality for individuals with type 2 diabetes mellitus (T2DM) [1, 2]. Common conditions coexisting with T2DM such as hypertension and dyslipidemia are clear risk factors for ASCVD [1, 2]. For the past decades, controlling multiple cardiovascular risk factors have shown the efficacy of reducing or slowing ASCVD in people with T2DM [3]. However, the risk of recurrent major cardiovascular events (CVEs) remains high despite the intensive statin treatment and other secondary prevention strategies were recommended [4, 5]. Therefore, searching potential risk factors contributing to this residual cardiovascular risk is crucial for improving the long-term prognosis in patients with T2DM and a first CVE.

Elevated lipoprotein(a) (Lp[a]) represents one of the most common genetic dyslipidemias worldwide, affecting 1 in 5 individuals [6]. Close attention to Lp(a), a particle containing of a low-density lipoprotein (LDL)-like particle bound to apolipoprotein(a), has emergingly been paid due to its pathogenic role in atherosclerosis and thrombosis formation [6]. Epidemiological and prospective data have suggested that a high level of Lp(a) is an independent risk factor for incident cardiovascular disease (CVD) [7, 8], particularly among those with DM [9, 10]. Simultaneously, in the secondary prevention setting, elevated Lp(a) values were also proved to be an independent predictor of CVEs in patients with established coronary artery disease (CAD) [11] or patients undergone percutaneous coronary intervention (PCI) [12, 13]. Data from our team also delivered that Lp(a) levels were strongly associated with the presence and severity of CAD in individuals with DM [14] and could predict higher risk of subsequent CVEs in stable CAD patients with DM or pre-DM [15]. However, it is currently undetermined whether Lp(a) plays a role in predicting recurrent CVEs in patients who had experienced prior CVEs [16, 17], and even more, there is no large-scale study specific to the T2DM population.

Therefore, in this prospective, observational cohort study, we, for the first time, investigated the predictive value of Lp(a) with recurrent worse outcomes in T2DM patients with prior CVEs.

## Materials and methods

### Study population

The study complied with the Declaration of Helsinki and was approved by the hospital’s ethical review board (Fu Wai Hospital & National Center for Cardiovascular Diseases, Beijing, China). All enrolled subjects provided informed written consent in the current study.

**Fig. 1:**
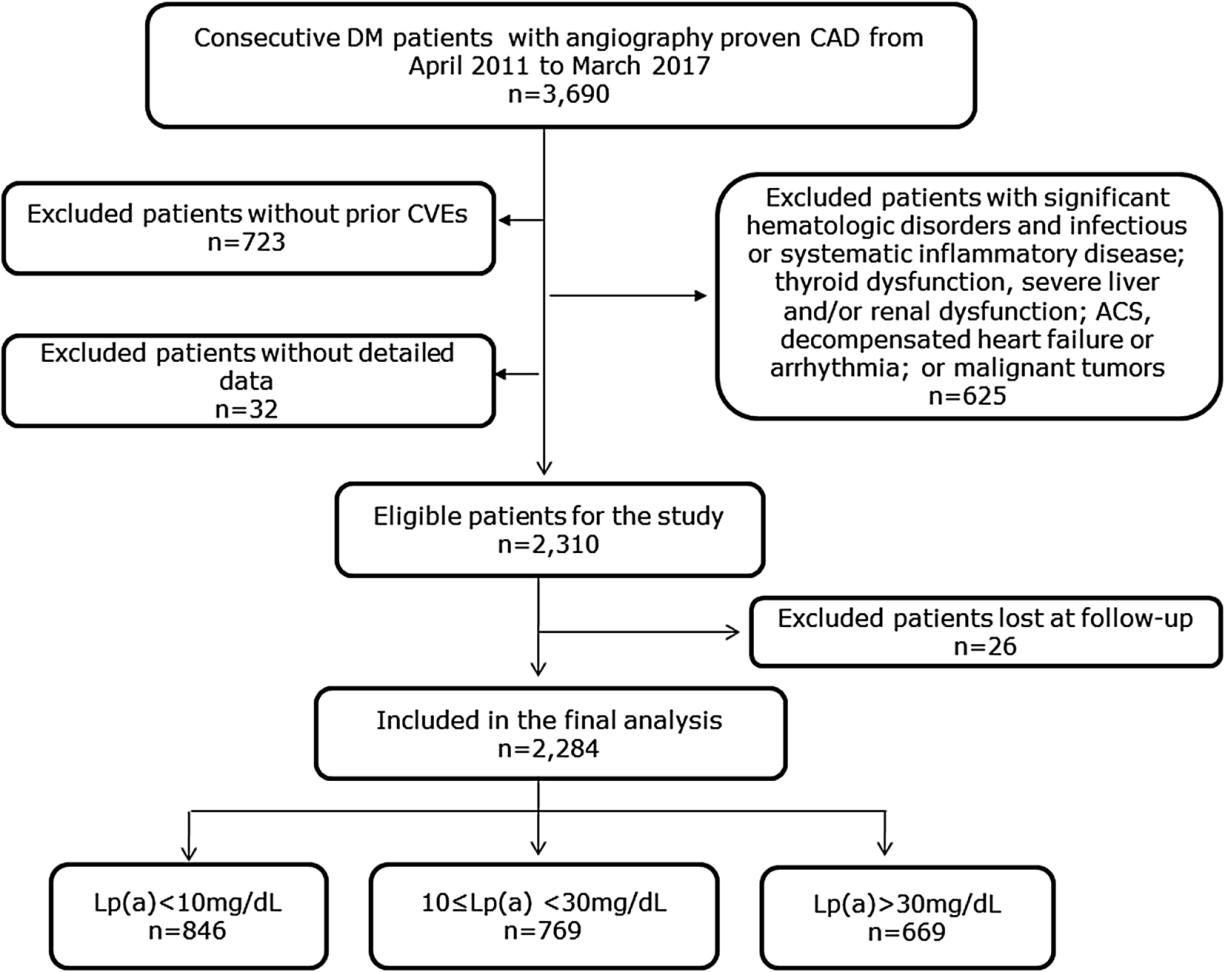
Flowchart of the enrolled study population

From April 2011 to March 2017 (as shown in Fig. 1), a total of 3690 T2DM patients with angiography proven stable CAD were consecutively recruited from three medical centers, including FuWai hospital, XuanWu Hospital, and AnZhen hospital according to the same protocol. The blood samples for testing Lp(a) were sent to FuWai hospital for unified measurement. The inclusion criteria were patients who had experienced a prior CVE [defined as myocardial infarction (MI), stroke, peripheral arterial disease, percutaneous coronary intervention (PCI), and coronary artery bypass grafting (CABG)] between 2 months to 1 year before admission. The exclusion criteria were patients with significant hematologic disorders and infectious or systematic inflammatory disease; thyroid dysfunction, severe liver and/or renal dysfunction; acute coronary syndrome (ACS), decompensated heart failure or arrhythmia; or malignant tumors, without detailed data. Finally, a total of 2310 eligible patients were enrolled in the current study. All study patients were prescribed secondary prevention medicine of ASCAD and followed up for adverse outcomes. Subsequently, a total of 26 patients were lost during the follow-up period. Therefore, there were 2284 T2DM patients with prior CVEs included in the final analysis, and were further divided into three groups according to Lp(a) levels.

### Follow-up

Patients were followed up at 6 months’ intervals through direct interviews or telephone by well-trained cardiologists or nurses who were blinded to the purpose of the study. The primary endpoints (recurrent CVEs) included cardiovascular death, non-fatal MI and stroke. For patients with suspected cardiovascular attacks, the medical records or emergency records were required to be sent to our centers. The endpoints were confirmed by at least two professional physicians.

### Definition of clinical status

The diagnosis of Nonfatal MI included ST-segment–elevation MI (STEMI) and non–ST-segment–elevation MI (NSTEMI). STEMI was defined as elevated biomarkers and new or presumed new ST-segment elevation in 2 or more contiguous leads. NSTEMI was defined as the presence of elevated biomarkers and at least 1 of either ECG changes (ST-segment depression or T-wave abnormalities), or ischemic symptoms. Stroke was diagnosed by the presence of typical symptoms and imaging. DM was diagnosed by fasting plasma glucose ≥ 7.0 mmol/L, the 2-h plasma glucose of the oral glucose tolerance test (OGTT) ≥ 11.1 mmol/L (based on venous plasma glucose results before and 2 h after a 75 g oral glucose load), or current use of hypoglycemic drugs or insulin. Hypertension was defined as repeated systolic blood pressure ≥ 140 mmHg or diastolic blood pressure ≥ 90 mmHg (at least two times in different environments) or currently taking anti-hypertensive drugs. Dyslipidemia was defined by medical history or fasting total cholesterol (TC) ≥ 5.18 mmol/L or triglyceride (TG) ≥ 1.7 mmol/L and/or high-density lipoprotein cholesterol (HDL-C) < 1.04 mmol/L (for male) or < 1.30 mmol/L (for female). Body mass index (BMI) was calculated as weight (kg) divided by height (m) squared. Overweight was defined as patients with BMI ≥ 25 kg/m^2^. Current smokers were defined as having smoked a cigarette in the past 30 days and > 100 cigarettes in a lifetime. Family history of CAD was defined as CAD occurring in a first-degree relative including mother, father, siblings, or child.

### Laboratory analysis

Blood samples were obtained from the cubital vein after at least 12 h of fasting in the current study. Concentrations of TC, TG, low density lipoprotein cholesterol (LDL-C), HDL-C levels were measured with an automatic biochemistry analyzer (7150; Hitachi, Tokyo, Japan) in an enzymatic assay. Apolipoprotein AI (apo AI), and apo B were measured by an immunoturbidimetric method (Tina-quant, Roche Diagnostics). Lp(a) was determined by immunoturbidimetry method [LASAY Lp(a) auto; SHIMA Laboratories Co., Ltd] with a normal value of < 30 mg/dL. An Lp(a) protein validated standard was used to calibrate the examination, and the coefficient of variation value of repetitive measurements was < 10%. The concentrations of glucose were measured by enzymatic hexokinase method, and HbA1c by a Tosoh Automated Glycohemoglobin Analyzer HLC-723G8.

### Statistical analysis

The data were expressed as the mean ± SD or median (Q1–Q3) for the continuous variables and the number (percentage) for the categorical variables. The Kolmogorov–Smirnov test was used to test the distribution pattern. The differences between continuous variables were determined with the Student’s *t* test, analysis of variance, Mann–Whitney U test, Kruskal–Wallis H test, and between the categorical variables were analyzed by χ2-test or Fisher’s exact test where appropriate. The event-free survival rates among groups were calculated by the Kaplan–Meier analysis and compared by the log-rank test. Univariate and multivariate Cox proportional hazard models were used to calculate the hazard ratio (HR) and 95% confidence interval (CI). To identify whether Lp(a) could improve the prediction of recurrent CVEs based on the SMART risk score model [18], we calculated Harrell’s C-statistic in the current analysis. A p-values of less than 0.05 were considered statistically significant. The statistical analyses were performed with SPSS version 22.0 software (SPSS Inc., Chicago, IL, USA) and R language version 3.6.3 (Feather Spray).

## Results

### Baseline characteristics

**Fig. 2:**
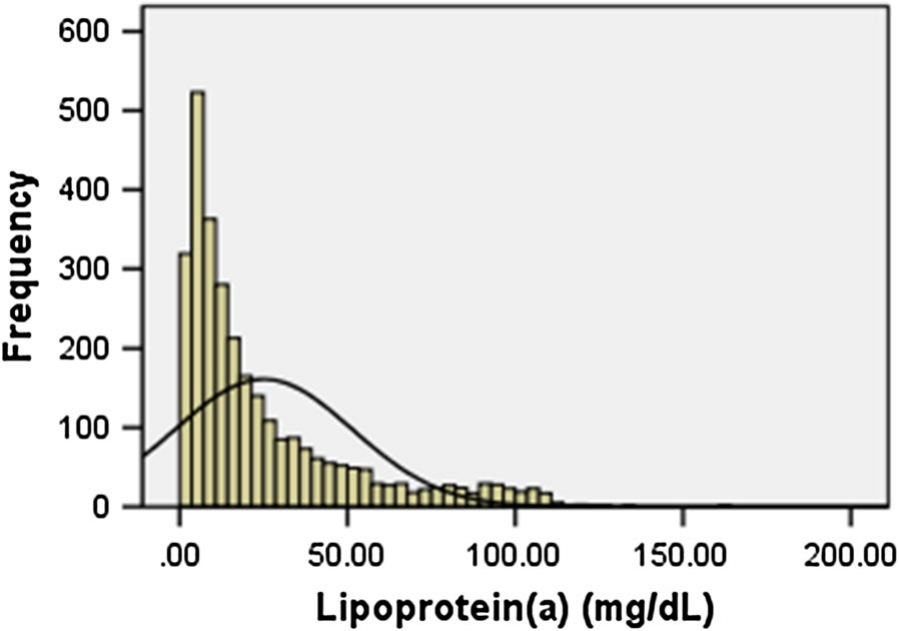
The distribution of lipoprotein(a) levels in patients with DM

Consistent with previous researches [19, 20], the plasma lipoprotein(a) levels had a skewed distribution in the overall 2284 enrolled population (as shown in Fig. 2). Table 1 summarizes study sample characteristics stratified by Lp(a) levels (Low: Lp[a] < 10 mg/dL, n = 846; Medium: 10 mg/dL ≤ Lp[a] < 30 mg/dL, n = 769; High: Lp[a] ≥ 30 mg/dL, n = 669). Mean age of study participants was 58.54 years and 73.3% were male. Most participants were considered to have traditional CVD risk factors including hypertension (69.6%), dyslipidemia (79.6%), and current smokers (57.4%), while only 13.7% of the enrolled patients have family history of CAD. Participants in the high Lp(a) group (Lp[a] ≥ 30 mg/dL) had less male patients, higher TC, LDL-C, apolipoprotein B levels, lower plasma TG levels, and tended to have more multi-diseased vessels.

**Table 1:** Baseline clinical characteristics of the study participants according to Lp(a) categories

Variables	All patients	Lp (a) categories (mg/dL)			p value
	(n = 2284)	< 10	10 ~ 30	≥30	
		(n = 846)	(n = 769)	(n = 669)	
Clinical characteristics					
Age, years	58.54 ± 10.47	57.99 ± 10.66	58.74 ± 10.61	59.00 ± 10.06	0.143
Male, n (%)	1674 (73.3)	663 (78.4)	550 (71.5)	461 (68.9)	**<***0.001*
Hypertension, n (%)	1589 (69.6)	607 (71.8)	529 (68.8)	453 (67.8)	0.199
Dyslipidemia, n (%)	1817 (79.6)	677 (80.1)	612 (79.7)	528 (79.0)	0.885
Current smokers, n (%)	1312 (57.4)	507 (59.9)	445 (57.9)	360 (53.8)	0.052
Family history of CAD, n (%)	312 (13.7)	103 (12.2)	109 (14.2)	100 (14.9)	0.281
Body mass index, kg/m^2^	26.35 ± 3.15	26.64 ± 3.12	26.18 ± 3.27	26.19 ± 3.02	*0.005*
SBP, mmHg	128 ± 17	128 ± 17	128 ± 18	127 ± 16	0.288
DBP, mmHg	78 ± 16	78 ± 11	78 ± 22	77 ± 11	0.133
Heart rate, bpm	71 ± 10	72 ± 10	71 ± 10	71 ± 11	0.163
Laboratory and clinical parameters					
FBG, mmol/L	7.24 ± 2.31	7.33 ± 2.35	7.23 ± 2.34	7.12 ± 2.24	0.220
HbA1c, %	7.39 ± 1.26	7.35 ± 1.22	7.44 ± 1.29	7.38 ± 1.28	0.311
TC, mmol/L	4.08 ± 1.18	3.97 ± 1.21	4.02 ± 1.10	4.29 ± 1.20	**< ***0.001*
HDL-C, mmol/L	1.01 ± 0.27	1.00 ± 0.27	1.01 ± 0.26	1.03 ± 0.28	0.054
LDL-C, mmol/L	2.45 ± 0.97	2.28 ± 0.92	2.42 ± 0.90	2.69 ± 1.06	**< ***0.001*
TG, mmol/L	1.56 (1.17–2.20)	1.65 (1.19–2.42)	1.54 (1.16–2.14)	1.48 (1.13–2.09)	**< ***0.001*
Lp (a), mg/dL	15.01 (6.60–34.76)	5.22 (3.32–7.33)	17.19 (13.11–22.54)	52.94 (38.85–79.41)	**< ***0.001*
ApoAI, g/L	1.31 ± 0.30	1.32 ± 0.35	1.29 ± 0.26	1.31 ± 0.29	0.179
ApoB, g/L	0.92 ± 0.30	0.87 ± 0.29	0.90 ± 0.28	0.99 ± 0.31	**< ***0.001*
Diseased vessels, n (%)					*0.016*
One vessel	430 (18.8)	174 (20.6)	151 (19.6)	105 (15.7)	
Two vessels	677 (29.6)	273 (32.3)	218 (28.4)	186 (27.8)	
Multi-vessels	1138 (49.8)	382 (45.1)	391 (50.8)	365 (54.5)	
LVEF, %	62.3 ± 8.7	62.3 ± 8.8	62.3 ± 9.1	62.2 ± 8.2	0.971
Medications					
Aspirin, n (%)	2227 (97.5)	827 (97.7)	753 (97.8)	647 (96.8)	0.418
P2Y12 inhibitor, n (%)	2040 (89.3)	750 (88.6)	703 (91.4)	587 (87.8)	0.070
Statins, n (%)	2133 (93.4)	783 (92.7)	730 (94.9)	620 (92.7)	0.150
ACEI/ARB, n (%)	1215 (53.2)	453 (53.6)	402 (52.3)	360 (53.8)	0.825
β-blockers, n (%)	1886 (82.6)	711 (84.0)	617 (80.2)	558 (83.4)	0.117
CCB, n (%)	875 (38.3)	335 (39.6)	295 (38.3)	245 (36.7)	0.513
Anti-diabetes treatment					0.757
Oral drugs	1311 (57.4)	488 (57.7)	435 (56.6)	388 (58.0)	
Insulin	726 (31.8)	259 (30.6)	254 (33.0)	213 (31.9)	

Continuous values are summarized as mean ± SD, median (Q1–Q3) and categorical variables as n (%)

*Lp(a)* lipoprotein(a), *CAD* coronary artery disease, *SBP* systolic blood pressure, *DBP* diastolic blood pressure, *FBG* fasting blood glucose, *HbA1c* glycosylated hemoglobin, *TC* total cholesterol, *HDL-C* high-density lipoprotein cholesterol, *LDL-C* low-density lipoprotein cholesterol, *TG* triglyceride, *ApoAI* apolipoprotein AI, *ApoB* apolipoprotein B, *LVEF* left ventricular ejection fraction, *ACEI* angiotensin converting enzyme inhibitors, *ARB* angiotensin receptor blockers, *CCB* calcium channel blockers

Of these, 1311 (57.4%) was only on oral anti-diabetes drugs, and 726 (31.8%) had insulin treatment. Meanwhile, there was no significant difference with regard to anti-diabetes drug therapy as well as prescribed secondary prevention medicines such as aspirin, P2Y12 inhibitor, statins, angiotensin converting enzyme inhibitor/angiotensin receptor blockers (ACEI/ARB), β-blockers, and calcium channel blocker (CCB) among groups (Table 1).

### Relation of risk factors and recurrent CVEs

Over 7613 patient-years’ follow-up period, 153 recurrent CVEs occurred (68 been identified as cardiovascular death, 30 suffered nonfatal MI, and 55 had strokes) as shown in Table 2. Patients with recurrent CVEs were much older, with lower percentage of overweight. Of note, the Lp(a) levels were dramatically higher in patients with recurrent CVEs compared with those without recurrent CVEs (20.44 mg/dL vs. 14.71 mg/dL, p = 0.002). Nevertheless, the gender, blood pressure, heart rate, fasting blood glucose, HbA1c, TC, HDL-C, LDL-C, TG, apo A1, and apo B were balanced between patients with or without recurrent CVEs (all p > 0.05). The association of exposure and other variables with recurrent CVEs in patients with T2DM were shown in Additional file 1: Table S2.

**Table 2:** Clinical and traditional risk factors in patients with and without recurrent CVEs

Characteristics	With recurrent CVEs (n = 153)	Without recurrent CVEs (n = 2131)	p value
Age, years	62.58 ± 9.17	58.25 ± 10.50	**< ***0.001*
Male, n (%)	115 (75.2)	1559 (73.2)	0.637
Body mass index, kg/m^2^	25.85 ± 3.09	26.39 ± 3.15	*0.042*
BMI < 25 kg/m2	70 (45.9)	718 (33.7)	*0.003*
SBP, mmHg	127 ± 18	128 ± 17	0.645
SBP < 130 mmHg	78 (51.2)	1057 (49.6)	0.785
DBP, mmHg	76 ± 10	78 ± 16	0.130
DBP < 80 mmHg	72 (47.3)	889 (41.7)	0.231
Heart rate, bpm	71 ± 10	71 ± 10	0.761
Biochemical parameters			
FBG, mmol/L	7.12 ± 2.43	7.24 ± 2.31	0.526
FBG 4.4 ~ 6.5 mmol/L	60 (38.9)	886 (41.6)	0.548
HbA1c, %	7.53 ± 1.37	7.38 ± 1.25	0.150
HbA1c < 7.0%	74 (48.4)	1072 (50.3)	0.932
TC, mmol/L	4.03 ± 1.08	4.08 ± 1.19	0.618
HDL-C, mmol/L	0.99 ± 0.26	1.01 ± 0.27	0.325
LDL-C, mmol/L	2.44 ± 0.93	2.45 ± 0.97	0.942
LDL-C < 1.4 mmol/L	16 (10.6)	232 (10.9)	0.907
TG, mmol/L	1.54 (1.17–2.12)	1.56 (1.17–2.20)	0.502
Lp(a), mg/dL	20.44 (10.01–43.96)	14.71 (6.43–34.16)	*0.002*
ApoAI, g/L	1.29 ± 0.30	1.31 ± 0.30	0.407
ApoB, g/L	0.91 ± 0.30	0.92 ± 0.30	0.746

Continuous values are summarized as mean ± SD, median (Q1–Q3) and categorical variables as n (percentage)

*CVEs* cardiovascular events, *SBP* systolic blood pressure, *DBP* diastolic blood pressure, *FBG* fasting blood glucose, *HbA1c* glycosylated hemoglobin, *TC* total cholesterol, *HDL-C* high-density lipoprotein cholesterol, *LDL-C* low-density lipoprotein cholesterol, *TG* triglyceride, *Lp(a)* lipoprotein(a), *ApoAI* apolipoprotein AI, *ApoB* apolipoprotein B

### Lp(a) levels and recurrent CVEs

**Fig. 3:**
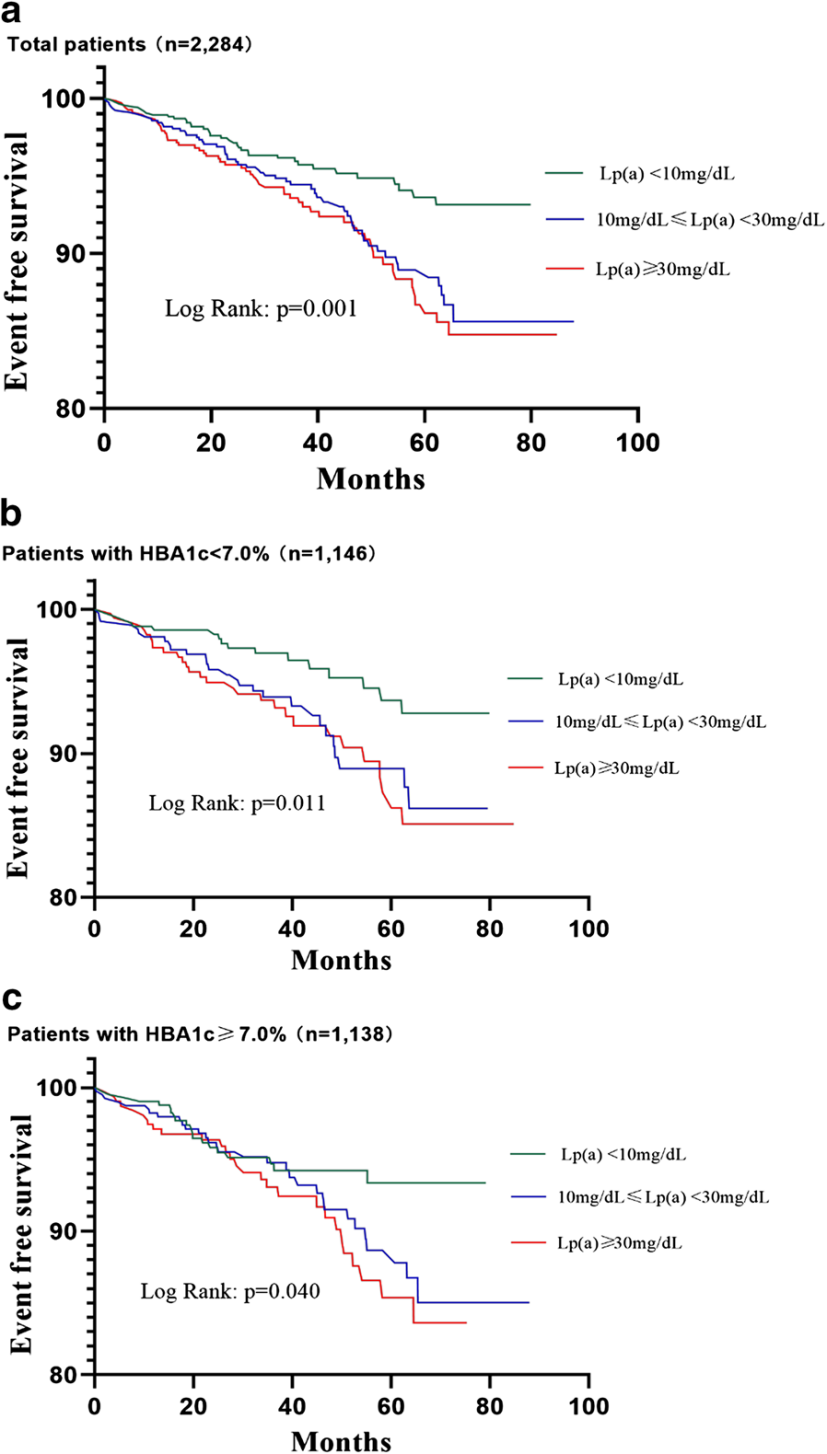
Kaplan-Meier analysis of Lp(a) categories for predicting Recurrent CVEs in subjects with different HBA1c status

The incidence of the composite recurrent CVEs in the low, medium, and high Lp(a) groups (based on the cut-off value of 10 and 30 mg/dL) was 4.4%, 7.7%, and 8.5%, respectively. As shown in Fig. 3a, the Kaplan–Meier analysis showed that subjects with medium and high Lp(a) value had a significantly lower cumulative event-free survival rate compared to those with low Lp(a) value (p = 0.001). Similar results were found in patients with HbA1c < 7.0% (p = 0.011, Fig. 3b) and HbA1c ≥ 7.0% (p = 0.040, Fig. 3c) group.

As presented in Table 3, univariate Cox regression models showed that the hazard ratio of recurrent CVEs in patients with medium and high Lp(a) was 1.736-fold, 1.960-fold higher than ones with low Lp(a) values. Additional adjustment for other variables did not change the significance of high Lp(a) with recurrent CVEs (HR 2.049 [95% CI 1.308–3.212], p = 0.002). When divided the composite recurrent CVEs into three separate endpoints including non-fatal MI, stroke, and cardiovascular death, high Lp(a) group had a 3.016-fold hazard ratio of non-fatal MI (p = 0.026), and a 2.708-fold hazard ratio of cardiovascular death (p = 0.006) compared with low Lp(a) group. However, high Lp(a) group did not have an increase in stroke risk compared with the low Lp(a) group (p = 0.777). Furthermore, the relationship of Lp(a) levels with recurrent CVEs did not impacted by HBA1c status (as indicated in Table 4, p < 0.05). In a sensitivity analysis by excluding  individuals with CABG, stroke and peripheral arterial disease (*n* = 1758), high Lp(a) remains an independent predictor of recurrent CVEs in this population (medium Lp(a) categories: HR 2.244 [95% CI 1.263–3.984], p = 0.006; high Lp(a) categories: HR 2.399 [95% CI 1.313–4.383], p = 0.004; respectively) after adjusting for potential confounding factors (shown in Additional file 1: Table S1).

**Table 3:** Relation of Lp(a) levels with composite and separate recurrent CVEs in patients with T2DM

Endpoints	Recurrent CVEs/Total	Crude model		Adjusted model	
		HR (95% CI)	p value	HR (95% CI)	p value
Composite recurrent CVEs	153/2284				
Lp(a) per-SD increase		1.007 (1.002–1.013)	*0.007*	1.008 (1.002–1.014)	*0.006*
Lp(a) < 10	37/846	Reference		Reference	
10 ≤ Lp(a) < 30	59/769	1.736 (1.151–2.619)	*0.009*	1.720 (1.099–2.692)	*0.018*
Lp(a) ≥ 30	57/669	1.960 (1.296–2.965)	*0.001*	2.049 (1.308–3.212)	*0.002*
Non-fatal MI	30/2284				
Lp(a) per-SD increase		1.012 (1.001–1.023)	*0.039*	1.012 (1.000–1.024)	*0.050*
Lp(a) < 10	7/846	Reference		Reference	
10 ≤ Lp(a) < 30	8/769	1.259 (0.457–3.472)	0.656	1.539 (0.529–4.474)	0.428
Lp(a) ≥ 30	15/669	2.737 (1.116–6.714)	*0.028*	3.016 (1.144–7.949)	0.026
Stroke	55/2284				
Lp(a) per-SD increase		1.002 (0.992–1.011)	0.761	1.001 (0.990–1.012)	0.815
Lp(a) < 10	17/846	Reference		Reference	
10 ≤ Lp(a) < 30	22/769	1.408 (0.748–2.651)	0.289	1.355 (0.674–2.724)	0.394
Lp(a) ≥ 30	16/669	1.199 (0.606–2.372)	0.603	1.118 (0.518–2.413)	0.777
CVD deaths	68/2284				
Lp(a) per-SD increase		1.009 (1.002–1.017)	*0.019*	1.011 (1.003–1.020)	*0.011*
Lp(a) < 10	13/846	Reference		Reference	
10 ≤ Lp(a) < 30	29/769	2.419 (1.257–4.652)	*0.008*	2.242 (1.108–4.535)	*0.025*
Lp(a) ≥ 30	26/669	2.539 (1.305–4.942)	*0.006*	2.708 (1.340–5.475)	*0.006*

The adjusted model including age, sex, body mass index, current smoking, hypertension, dyslipidemia, family history of coronary artery disease, diseased vessels, low-density lipoprotein cholesterol, fasting blood glucose, statin and anti-diabetes drugs use

*CVEs* cardiovascular events, *MI* myocardial infarction, *CVD* cardiovascular disease

**Table 4:** Association of Lp(a) levels with recurrent CVEs in T2DM patients according to HBA1c status

Lp(a) (mg/dL)	Recurrent CVEs/Total	Crude model		Adjusted model	
	(153/2284)	HR (95% CI)	p value	HR (95% CI)	p value
Total patients	153/2284				
Lp(a) per-SD increase		1.007 (1.002–1.013)	*0.007*	1.008 (1.002–1.014)	*0.006*
Lp(a) < 10	37/846	Reference		Reference	
10 ≤ Lp(a) < 30	59/769	1.736 (1.151–2.619)	*0.009*	1.720 (1.099–2.692)	*0.018*
Lp(a) ≥ 30	57/669	1.960 (1.296–2.965)	*0.001*	2.049 (1.308–3.212)	*0.002*
HBA1c < 7.0%	74/1146				
Lp(a) per-SD increase		1.008 (1.001–1.016)	*0.024*	1.009 (1.001–1.018)	*0.023*
Lp(a) < 10	17/427	Reference		Reference	
10 ≤ Lp(a) < 30	28/373	1.968 (1.077–3.595)	*0.028*	1.815 (0.949–3.473)	0.072
Lp(a) ≥ 30	29/346	2.156 (1.185–3.924)	*0.012*	2.009 (1.051–3.840)	*0.035*
HBA1c ≥ 7.0%	79/1138				
Lp(a) per-SD increase		1.006 (0.998–1.014)	0.122	1.009 (1.000–1.018)	0.050
Lp(a) < 10	20/418	Reference		Reference	
10 ≤ Lp(a) < 30	31/402	1.520 (0.866–2.667)	0.144	1.571 (0.842–2.932)	0.155
Lp(a) ≥ 30	28/318	1.816 (1.023–3.223)	*0.042*	2.162 (1.148–4.073)	*0.017*

The adjusted model including age, sex, body mass index, currentis smoking, hypertension, dyslipidemia, family history of coronary artery disease, diseased vessels, low-density lipoprotein cholesterol, fasting blood glucose, statin and anti-diabetes drugs use

*CVEs* cardiovascular events, *MI* myocardial infarction, *CVD* cardiovascular disease

### Risk prediction for recurrent CVEs

As presented in Table 5, in the whole population, Cox prediction using the SMART risk score model, the C-statistic values were 0.660 (95% CI 0.595–0.726). Furthermore, adding Lp(a) categories to the original model resulted in a significant improvement in C-statistic (ΔC-statistic 0.029 [0.006–0.062], p = 0.047).

**Table 5:** C-statistic of Lp(a) categories for predicting recurrent CVEs

Models	C-statistic (95% CI)	ΔC-statistic (95% CI)	p value
Total patients (n = 2284)			
Original model	0.660 (0.595–0.726)	Reference	
Original model + Lp(a) categories	0.689 (0.625–0.753)	0.029 (0.006–0.062)	*0.047*

Original model was using the SMART risk score model

## Discussion

Our study enrolled a prospective cohort corresponding to diabetic individuals with prior established CVEs, who were at high risk for recurrent ischemic CVEs in the circumstance of following standard secondary prevention strategies recommended by the current guidelines [21, 22]. Data, for the first time, clearly confirmed that Lp(a) was an independent predictor for recurrent CVEs in T2DM patients with prior CVEs. When stratified by HBA1c levels (< 7.0%, or ≥ 7.0%), this association were significant in both HBA1c status independent of the level of the other risk factors. More importantly, in the overall cohort, the addition of Lp(a) to the model improved the risk prediction for recurrent CVEs. Thus, the present study strongly implied that Lp(a) might be a useful marker for further risk stratification in patients with T2DM after they suffered a first CVE.

The prevalence of T2DM has been increasing dramatically over the past few decades, with projections of an even greater growth over coming decades [23, 24]. Convincing evidence indicated that CAD is a common comorbidity in patients with T2DM and has been considered as a CAD risk equivalent based on multiple guidelines [25]. Currently, several clinical investigations indicated that despite aggressive multidisciplinary efforts have been made including revascularization and intensive management of LDL-C, glucose, blood pressure, and thrombotic risk, patients surviving an ACS event are at increased risk of recurrent CVEs, and this risk is further increased in patients with T2DM [26], raising the question of whether the treatment regimens are less effective in these patients. For decades, it has been well elucidated that abnormal lipid metabolism largely contributes to the additional cardiovascular risk for T2DM patients [27]. Therefore, the management of multiple risk factors especially lipid is of great significance for the prognosis. The recent guidelines have clearly recommended the target value of LDL-C [28], nonetheless, residual cardiovascular risk remains high for T2DM patients with a prior CVE compared with non-diabetic patients. Thus, it is essential to search additional modifiable lipid disorders to further improve the prognosis of these patients. Therefore, we consecutively recruited 2284 T2DM patients with prior CVEs and followed up for 7613 patient-years, attempting to seek plausible residual risk in terms of lipid disorders.

Recently, the relationship of elevated Lp(a) with CVD risk have been emergingly recognized in multiple investigations. Plasma concentrations of Lp(a) are mainly (90%) determined by the LPA gene, without significant dietary or environmental influences [29]. The association of Lp(a) with risk of CAD as well as mortality, which is independent of traditional cardiovascular risk factors, has been rapidly aware in series of studies [7, 30]. Lp(a) has been determined as the independent genetic risk factor of CVD and a causal role has been demonstrated by Mendelian randomization [31]. The Copenhagen City Heart Study demonstrated that compared to subjects with Lp(a) levels below 5 mg/dL, those with Lp(a) between 30 and 76 mg/dL had a 1.6-fold increased risk for incident MI. This risk increased to 1.90 for individuals with Lp(a) between 77 and 117 mg/dL and to 2.60 for individuals with Lp(a) concentrations above 117 mg/dL [8]. However, the data mainly based on investigations of apparently healthy participants in the general population rather than patients with a prior CVE. At the same time, among limited existing investigations related to patients with established CAD, inconsistent results were also observed. A recent cohort study support that in patients with stable CAD and chronic total occlusion, increased Lp(a) confers greater risk for poor coronary collateralization when TC, LDL-C or non-HDL-C are elevated especially in patients with T2DM [32]. In the previous study involving stable CAD patients with different glucose metabolism status, high Lp(a) were associated with significantly higher risk of subsequent CVEs in pre-DM and DM [15]. However, the enrolled population were restricted to patients with stable CAD but not those with prior CVEs. On the contrary, Schwartz GG, et al. enrolled 969 patients who experienced a recent ACS and treated with statins, Lp(a) concentration was not associated with adverse CVEs [16]. Additionally, the study conducted by Gencer et al. also suggested that high Lp(a) levels are not predictive for cardiovascular outcomes in patients otherwise medically well controlled, but might be useful to identify patients who would not be on LDL‐C targets 1 year after ACS [33]. The above two studies were localized in patients with the acute setting of ACS, and could not reflect the situation of DM with previous ASCVD attack. Therefore, studies concerning the prognosis of Lp(a) in patients with a prior CVE are of worth in the real-world, particularly in patients with T2DM.

Consequently, in our study, we observed that Lp(a) levels were significantly higher in patients suffered recurrent CVEs. Of note, our current data also demonstrated that the event-free survival rate was dramatically lower in medium and high Lp(a) groups. Significantly, compared with patients with low Lp(a) levels, those with high Lp(a) had a 2.049-fold higher hazard ratio of recurrent CVEs after adjusting for other variables including LDL-C, HBA1c, and so forth. Furthermore, when divided the population into two groups by HBA1c status, the predictive value of Lp(a) in risk of recurrent CVEs remains significant independent of the glucose control level. Finally, the C-statistic was significantly improved by 0.029 when added Lp(a) to the Cox model. Although the results were inconsistent with the study conducted by Schwartz GG [16], the different of enrolled population may partly explain the disparity. As far as we know, it is the first large study involved T2DM patients with a first CVE, which included the composite of MI, stroke, peripheral arterial disease, PCI, and CABG, instead of ACS or other specific status. Hence, the present study supported the opinion that Lp(a) was an independent predictor for recurrent CVEs in T2DM patients with prior CVEs in the stain era.

Till now, the mechanisms of Lp(a) potentiates CVD risk can be broadly classified in 3 categories: proatherogenic, proinflammatory, and potentially antifibrinolytic [29]. However, the exact mechanism of Lp(a) increasing CVD risk in DM status was not well clarified. The recent study assessed the relevance of biomarkers combined to pathway groups for the development of T2DM and coronary heart disease (CHD) during the median of 14 years follow‑up. The authors finally demonstrated that Lp(a) was inversely associated with T2DM and positively with CHD development [34]. However, the potentially causal mechanisms for both diseases, especially in relation to the observed opposite effect directions, are currently still obscure. More investigations were needed in the future.

Nevertheless, our study had several limitations. First of all, this is a study among Chinese population with T2DM and prior CVEs, and whether the data applied to other populations need to be testified. Secondly, the Lp(a) concentrations were only measured at baseline, and the alterations of the biomarkers may also be clinically significant during the follow-up period. Moreover, the method of Lp(a) measurement used in the study might be influenced by the apo(a) size due to the numbers of the KIV type 2 domain. Variations of apo(a) size between assay calibrators and patients’ samples might overestimate or underestimate the real concentration of Lp(a). Finally, as this was an observational study, further investigations are needed to clarify the underlying mechanism of the associations.

## Conclusions

Our data for the first time indicated that Lp(a) was an independent predictor for recurrent CVEs in T2DM patients with prior CVEs, suggesting that Lp(a) measurement may help further risk stratification for T2DM patients after they suffered a first CVE.

## Supplementary information

**Additional file 1.** Additional tables. **Table S1.** Relation of Lp(a) levels with recurrent CVEs in T2DM patients without CABG, peripheral arterial disease, and stroke. **Table S2.** Association of exposure and other variables with recurrent CVEs in patients with T2DM.

## Data Availability

The datasets used and/or analyzed during the current study are available from the corresponding author on reasonable request.

## Abbreviations

ACS: Acute coronary syndrome
ASCVD: Atherosclerotic cardiovascular disease
CABG: Coronary artery bypass grafting
CAD: Coronary artery disease
CVD: Cardiovascular disease
CVEs: Cardiovascular events
HDL-C: High-density lipoprotein cholesterol
LDL-C: Low-density lipoprotein cholesterol
Lp(a): Lipoprotein(a)
MI: Myocardial infarction
PCI: Percutaneous coronary intervention
TC: Total cholesterol
TG: Triglyceride
