# Supplementary material for: Lipoprotein (a) predicts recurrent worse outcomes in type 2 diabetes mellitus patients with prior cardiovascular events: a prospective, observational cohort study
Source: Cardiovasc Diabetol. 2020 Jul 9;19:111. doi: 10.1186/s12933-020-01083-8 (PMC7350185; doi:10.1186/s12933-020-01083-8)
Supplement: Supplementary file 1 — Additional file 1. Additional tables. Table S1. Relation of Lp(a) levels with recurrent CVEs in T2DM patients without CABG, peripheral arterial disease, and stroke. Table S2. Association of exposure and other variables with recurrent CVEs in patients with T2DM. [file 12933_2020_1083_MOESM1_ESM.docx]

**Additional Tables**

**Additional Table S1.** Relation of Lp(a) levels with recurrent CVEs in T2DM patients without CABG, PAD, and stroke

| Endpoints | Recurrent CVEs/Total | Crude model | | Adjusted model | |
| --- | --- | --- | --- | --- | --- |
|  |  | HR (95% CI) | p value | HR (95% CI) | p value |
| **Composite recurrent CVEs** | 95/1,758 |  |  |  |  |
| Lp(a) per-SD increase | | 1.006(1.000-1.013) | **0.050** | 1.008(1.000-1.016) | **0.042** |
| Lp(a) <10 | 20/665 | Reference |  | Reference |  |
| 10≤Lp(a) <30 | 42/600 | 2.292(1.346-3.904) | **0.002** | 2.244(1.263–3.984) | **0.006** |
| Lp(a)≥30 | 33/493 | 2.222(1.275-3.873) | **0.005** | 2.399(1.313-4.383) | **0.004** |

The adjusted model including age, sex, body mass index, current smoking, hypertension, dyslipidemia, family history of coronary artery disease, diseased vessels, low-density lipoprotein cholesterol, fasting blood glucose, statin and anti-diabetes drugs use. CVEs, cardiovascular events; Lp(a), lipoprotein(a).

**Additional Table S2.** Association of exposure and other variables with recurrent CVEs in patients with T2DM

| Variables | HR (95% CI) | p value |
| --- | --- | --- |
| sex | 0.899(0.623-1.297) | 0.568 |
| age | 1.036(1.020-1.053) | **<0.001** |
| Body mass index | 0.950(0.902-1.002) | **0.058** |
| Hypertension | 0.755(0.523-1.089) | 0.133 |
| Dyslipidemia | 1.191(0.816-1.736) | 0.365 |
| Current smokers | 0.855(0.619-1.182) | 0.344 |
| Family history of CAD | 0.878(0.589-1.309) | 0.523 |
| Diseased vessels | 1.35(1.077-1.692) | **0.009** |
| Antidiabetes drugs use | 1.019(0.808-1.284) | 0.876 |
| Statins use | 0.781(0.345-1.770) | 0.554 |
| Systolic blood pressure | 0.999(0.989-1.009) | 0.867 |
| Glucose | 1.013(0.944-1.087) | 0.723 |
| HBA1C | 1.097(0.972-1.239) | 0.135 |
| LDL-C | 0.985(0.833-1.165) | 0.860 |
| Lp(a) | 1.007(1.002-1.013) | **0.007** |
| Lp(a) categories |  |  |
| Lp(a) <10 | Reference |  |
| 10≤Lp(a) <30 | 1.736(1.151-2.619) | **0.009** |
| Lp(a)≥30 | 1.960(1.296-2.965) | **0.001** |

CAD, coronary artery disease; HbA1c, glycosylated hemoglobin; LDL-C, low-density lipoprotein cholesterol; Lp(a), lipoprotein(a).
